# Prevalence of mpox viral DNA in cutaneous specimens of monkeypox-infected patients: a systematic review and meta-analysis

**DOI:** 10.3389/fcimb.2023.1179885

**Published:** 2023-06-29

**Authors:** Isha Rani, Anmol Goyal, Muhammad Aaqib Shamim, Prakasini Satapathy, Amit Pal, Rosanna Squitti, Kalyan Goswami, Ranjit Sah, Joshuan J. Barboza, Bijaya K. Padhi

**Affiliations:** ^1^ Department of Biochemistry, Maharishi Markandeshwar College of Medical Sciences and Research (MMCMSR), Sadopur, Ambala, India; ^2^ Department of Community Medicine, Maharishi Markandeshwar College of Medical Sciences and Research (MMCMSR), Sadopur, Ambala, India; ^3^ Department of Pharmacology, All India Institute of Medical Sciences, Jodhpur, Rajasthan, India; ^4^ Department of Virology, Postgraduate Institute of Medical Education and Research, Chandigarh, India; ^5^ Department of Biochemistry, All India Institute of Medical Sciences (AIIMS), Kalyani, India; ^6^ Department of Laboratory Science, Research and Development Division, Fatebenefratelli Isola Tiberina, Gemelli Isola, Rome, Italy; ^7^ Department of Microbiology, Tribhuvan University Teaching Hospital, Institute of Medicine, Kathmandu, Nepal; ^8^ Department of Microbiology, Dr. D.Y Patil Medical College, Hospital and Research Centre, Pune, Maharashtra, India; ^9^ Department of Public Health Dentistry, Dr. D.Y. Patil Dental College and Hospital, Dr. D.Y. Patil Vidyapeeth, Pune, Maharashtra, India; ^10^ Escuela de Medicina, Universidad Cesar Vallejo, Trujillo, Peru; ^11^ Department of Community Medicine and School of Public Health, Postgraduate Institute of Medical Education and Research, Chandigarh, India

**Keywords:** monkeypox, mpox viral DNA, skin lesion, cutaneous, meta-analysis, infectivity potential, transmission

## Abstract

**Background:**

Human monkeypox (mpox) disease is a multicountry outbreak driven by human–human transmission which has resulted in an international public health emergency. However, there is limited evidence on the positivity rate of skin lesions for mpox viral DNA. We aim to fill this gap by estimating the pooled positivity rate of skin samples with mpox viral DNA from mpox patients globally.

**Methods:**

In this systematic review and meta-analysis, seven databases and several preprint servers have been extensively searched until 17 January 2023 according to a prospectively registered protocol (PROSPERO: CRD42023392505). Articles including the positivity rate of skin samples with mpox viral DNA in mpox-confirmed patients were considered eligible. After a quality assessment, a random-effect meta-analysis was used for pooled prevalence. To explore and resolve heterogeneity, we used statistical methods for outlier detection, influence analysis, and sensitivity analysis.

**Findings:**

Among the 331 articles retrieved after deduplication, 14 studies were finally included. The pooled positivity rate of the skin samples was 98.77% (95% CI: 94.74%–99.72%). After the removal of an influential outlier, *I* ^2^ for heterogeneity dropped from 92.5% to 10.8%. Meta-regression did not reveal any significant moderator.

**Conclusion/interpretation:**

The present findings reinforce that skin lesions act as a reservoir of mpox viral DNA and contribute to a high infectivity risk. This may be a prevailing basis of prompt transmission during the current multicountry outbreak and also needs further investigation. The present imperative outcome may benefit in producing valuable preventive and management procedures in an appropriate health strategy.

## 1 Introduction

Previous decades have witnessed multiple outbreaks of mpox (formerly known as monkeypox) infection in the Democratic Republic of the Congo (DRC), Nigeria, and Gambia (Ladnyj et al., 1972; MacNeil et al., 2009). With the growing multinational outbreak, the World Health Organization (WHO) declared mpox disease a potential “public health emergency of international concern (PHEIC)” on 23 July 2022 (World Health Organization (WHO), 2022a). According to Centers for Disease Control and Prevention (CDC) data, 85,922 cases of mpox and 96 deaths have been diagnosed globally since 1 February 2023. So far, mpox cases have been reported in 110 member states of all six WHO regions (CDC, 2023).

The mpox virus is a double-stranded DNA virus and belongs to an Orthopoxvirus genus of the Poxviridae family that includes the smallpox virus (MacNeil et al., 2009). The clinical manifestations of mpox are analogous to smallpox, but it is commonly less severe (Rubins et al., 2011). Even though mpox is a rare disease, it has a devastating impact on infected individuals. Furthermore, no suitable diagnostic test, precise therapy, or vaccine is still available (Shamim et al., 2023b). The clinical patterns observed in the current outbreak are different from the earlier African outbreaks. The clinical manifestations of mpox infection are mostly characterized by headaches, fever, chills, fatigue, myalgia, flu-like symptoms, skin lesions/rash, and lymphadenopathy. However, in the recent outbreak, atypical patterns of clinical symptoms have been reported in many cases (Huhn et al., 2005; Satapathy et al., 2022; Thornhill et al., 2022a; Gandhi AP. et al., 2023; Gandhi PA. et al., 2023). Most patients with moderate mpox do not require antiviral therapy or hospitalization (Huhn et al., 2005; Adler et al., 2022; Català et al., 2022). Importantly, the spread of the mpox virus occurs by direct or indirect close contact via sores, scabs, respiratory droplets or body fluids, and possibly contaminated surfaces or fomites (Thornhill et al., 2022a).

Indeed, international agencies and organizations are deeply concerned about the current epidemic. The effects of the transmission are alarming and staggering. Globally, future outbreaks may cause more severe mortality, morbidity, and broad economic impacts. Therefore, it is crucial to identify the route of transmission of the infection to implement approaches for empowering health strategies and social and environmental interventions.

In this regard, the positivity rate of viral particles in biological samples may provide an estimate of infectivity potential. A recent meta-analysis study revealed that skin lesions are the dominant clinical features of the current mpox outbreak (Liu et al., 2023). Consistently, other studies have evaluated the viral burden in skin samples of mpox patients (Peiró-Mestres et al., 2022; Thornhill et al., 2022a; Palich et al., 2023) and suggested that it increases with the severity of the disease (Huhn et al., 2005; Hennessee et al., 2022). According to these data, viral contents may be higher from skin lesions and predict the risk and severity of mpox infection. Validating if the prevalence of mpox viral DNA predicts the disease severity and infectivity might lead to treatment development, set up schemes, or even put in place activities to control community transmission. Additionally, cutaneous specimens are more easily accessible and minimally invasive than other biological specimens and, hence, are considered more suitable for diagnostic and prognostic purposes.

Keeping this in view, we performed a systematic review and meta-analysis of articles published till 17 January 2023 on the frequency of positive cutaneous specimens with mpox viral DNA of mpox patients. The results of this study can afford valuable insights into the illness and progress development of effective actions to restrain the spread of infection. Importantly, this novel information may be favorable for applying appropriate social measures to curtail the spread of the endemic infection.

## 2 Methods

This systematic review and meta-analysis is in accordance with the Meta-analysis of Observational Studies in Epidemiology (MOOSE) reporting guidelines (Stroup, 2000) and the 2020 Preferred Reporting Items for Systematic Reviews and Meta-Analyses (PRISMA) statement (Page et al., 2021). The review protocol was registered on the PROSPERO International Prospective Register of Systematic Reviews (CRD42023392505).

### 2.1 Search strategy

This systematic and meta-analysis search strategy was designed according to PECOS criteria (refer to **Supplementary Annex 2**) with the research question “What is the prevalence (or positivity rate; %) of skin samples with viral DNA in mpox patients.” Seven databases, namely, the Cochrane Library, EBSCOhost, EMBASE, ProQuest, PubMed/MEDLINE, Scopus, and Web of Science were searched for eligible articles till 17 January 2023 according to PECOS criteria (see **Supplementary Annex 2** for the search strategy and **Annex 1** for the PECOS criteria). A search strategy was prepared (IR) for PubMed with truncations, Boolean operators, and Medical Subject Heading (MeSH) terms. This was peer-reviewed by a second co-author (AG) in accordance with the Peer Review of Electronic Search Strategies: 2015 Guideline Statement (McGowan et al., 2016). The following terms were used: (mpox OR monkeypox OR mpxv) AND (skin OR cutaneous*) AND (lesion* OR swab OR sample). In addition, preprint servers (bioRxiv and medRxiv) were also examined to detect potentially eligible articles. This approach was further combined with manual exploration of citations in relevant articles, alongside checking forward citations. Google/Google Scholar was also searched for supplementary studies overlooked during the automated search.

### 2.2 Inclusion and exclusion criteria

The inclusion criteria were as follows: cases with mpox virus infection confirmed by real-time polymerase chain reaction (PCR). The confirmed cases were selected regardless of age, ethnicity, and gender. Observational studies such as cross-sectional, cohort, and case series published till 17 January 2023 were included in this study (**Supplementary Annex 1**). It is significant to indicate that relevant reports, communications, and editorials that provided the positivity rate of skin samples were also considered. Also, the exclusion criteria were as follows: suspected or probable subjects with mpox infection. Any irrelevant studies, abstracts, qualitative, randomized controlled trials (RCTs), policy, case reports, reviews, opinion reports, and articles without available full texts were excluded.

### 2.3 Selection criteria

All articles resulting from the electronic search were further imported into the reference management tool (Mendeley desktop V1.19.5) to manage the references and coordinate the review process. Furthermore, duplicate documents were eliminated by software function and also by manual reading of the title, authors, and journal name (IR and AG). Moreover, three randomized controlled trials (RCTs) from the electronic search were also removed. Clinical studies regarding mpox disease were separated by two authors (IR and AG) independently by reading the titles and abstracts of acquired studies by applying the eligibility criteria, and they selected 17 articles for full-text screening. Those articles concerning the prevalence of mpox DNA in cutaneous samples were selected by further reading the full text (IR and AG). Any differences between the two researchers (IR and AG) during the screening process were resolved through communication to preserve synchronization and decided consistently on the eligibility. The third author (MAS) decided on the unresolved doubts.

### 2.4 Data extraction and management

Two authors (IR and AG) independently extracted data from literature information in a Microsoft Excel spreadsheet, and any inconsistency at any stage was resolved by the authors through negotiation and discussion to build harmony. The third author (MAS) decided on the unsettled doubts. The subsequent information extracted from each of the included studies is given as follows: bibliographic details of the reports, characteristics of the study (study design, region where the study was conducted), characteristics of the participants (number of mpox confirmed cases from whom skin specimens were taken, age, gender), and summary measures (% of skin samples positive for mpox DNA).

**Figure 1:**
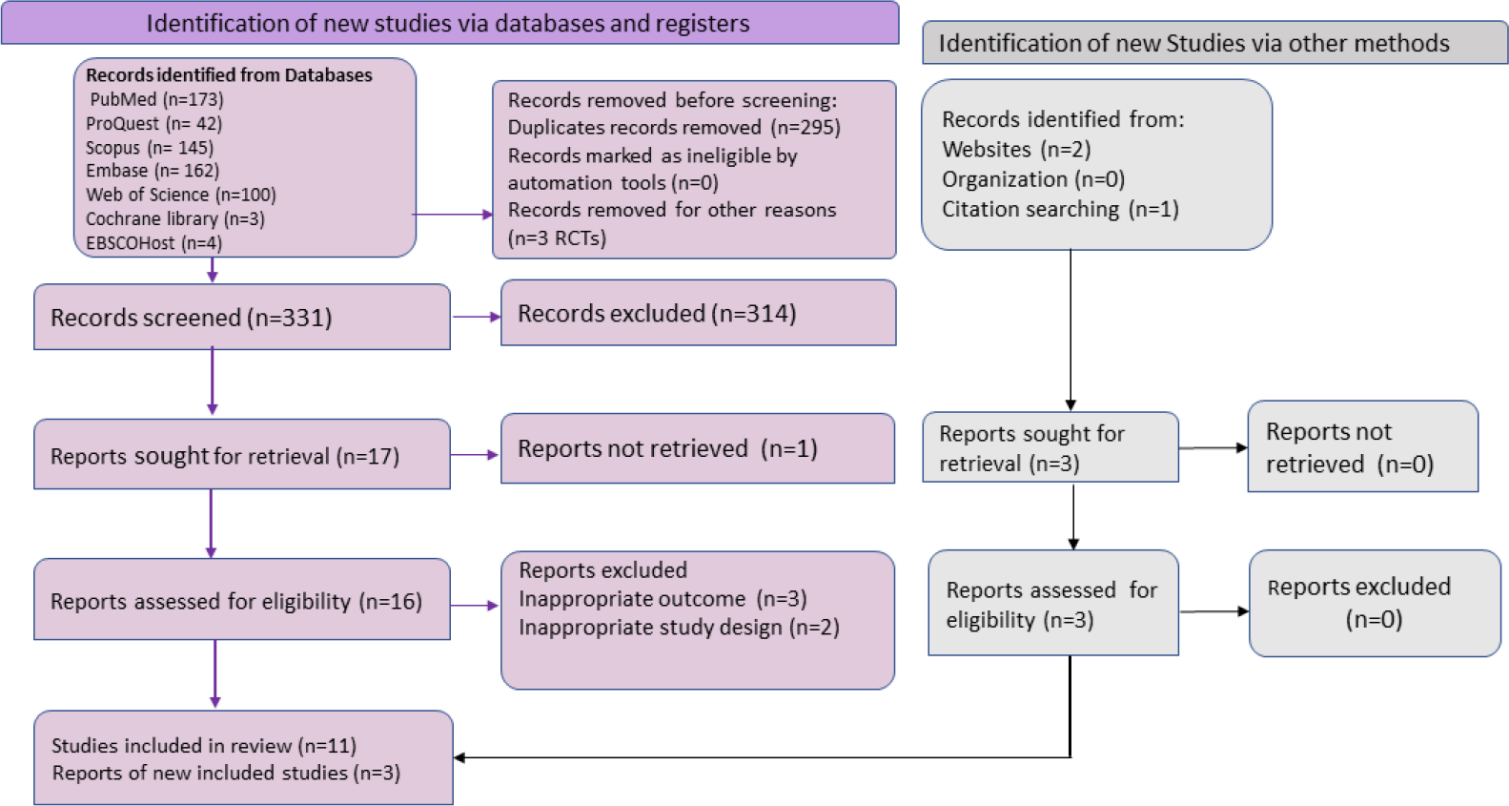
The Preferred Reporting Items for Systematic Reviews and Meta-Analyses (PRISMA) flowchart summarizing the literature search and giving reasons for the exclusion of studies.

The entire process of literature examination, screening, data extraction, systematic review, and meta-analysis was explained using the Preferred Reporting Standard of Systematic Reviews and Meta-Analyses (PRISMA-2020) flowchart and checklist to certify scientific precision (**Figure 1**).

### 2.5 Quality assessment

Two authors (IR and AG) independently assessed the risk of bias in the included literature via the quality assessment tools suggested by the National Institutes of Health (NIH) (National Institute of Health (NIH), 2021). The case series, cross-sectional, and cohort studies were evaluated with the NIH quality assessment tool. Any disparity between the authors (IG and AG) concerning the risk of bias in any of the studies was resolved by discussion. The third author (MAS) settled the unexplained ambiguities. The overall and rating scores for each study are explained in **Supplementary Annex 3**.

### 2.6 Statistical analysis

In the included studies, we extracted data on the percentage of mpox patients (diagnosed via any sample) whose skin samples also tested positive for mpox DNA. Heterogeneity was assessed using *I* ^2^, *H*, *τ* ^2^, and Cochran’s *Q*, apart from the prediction interval (Begg and Mazumdar, 1994; Higgins and Thompson, 2002). Prediction interval helps predict the effect size in a future study and does not merely provide the average effect across the available studies (Spineli and Pandis, 2020). It has been estimated based on a t-distribution. The choice of a fixed-effects model or a random-effects model is made depending upon factors including the observed heterogeneity. For synthesizing the results, a random intercept logistic regression model with logit transformation of proportions has been used.

In case of high heterogeneity as we encountered here, we will explore the cause behind heterogeneity and try to resolve or reduce it. Outlier detection will be done. Next, to detect influence, we will run a chain of statistical methods including the Baujat plot, influence diagnostics, leave-one-out meta-analyses, and graphical display of heterogeneity (GOSH) plots. We will also perform meta-regression using sample size and the average age of the participants as moderators (Sterne and Egger, 2005). This will be reported by the omnibus test of moderators employing a mixed-effects model and depicted visually using bubble plots. Publication bias and small-study effects will be assessed using the Doi plot and LFK index as these have been shown to be better suited for the meta-analysis of proportions (Furuya-Kanamori et al., 2018; Shamim et al., 2023a). All statistical analyses were performed using meta and metafor packages in the R programming language (v4.2.2) (R Core Team, 2020). The distribution of true effect size computations was carried out using Comprehensive Meta-Analysis Version 4 (Borenstein et al., 2022). A *p*-value <0.05 (two-sided) was considered statistically significant.

## 3 Results

### 3.1 Selection criteria

We identified 629 possibly relevant articles from the systematic search, among which 295 overlapping articles and 3 RCTs were excluded. After title and abstract screening of 331 articles, we retrieved 17 articles for the further review process. Out of 17, full-text screening was performed on 16 reports, while the full text of one article was not available and was eliminated (Gaspari et al., 2023). During the full-text screening, five articles did not fulfil the inclusion criteria and, thus, were not considered. Additionally, three other articles were also considered to be eligible as per inclusion criteria through related bibliography of the included reports and websites. Finally, a total of 14 studies were included in the meta-analysis for an overall pooled proportion (%) of mpox viral DNA in skin specimens (**Table 1**). The selection of literature is illustrated in the PRISMA flowchart (**Figure 1**). All 14 included studies were of good quality. The quality assessment of the included studies is depicted in the supplementary data (**Supplementary Annexs 3A, B**).

**Table 1:** Baseline characteristics of the included studies that reported the frequency of skin samples positive with mpox viral DNA [based on positivity rate (%)] in mpox patients (*n* = 14).

Authors (YOP)	Study design	Number of mpox confirmed cases from whom skin samples were taken	Outcome measure: frequency of positive skin samples with mpox viral DNA (%)	Age (years) (median)	Gender distribution	Geographical region
García-Piqueras P et al. (2022) (García-Piqueras et al., 2022)	C/Sl	53	100%	36	52 males including MSM or homosexuals and 1 female	Madrid, Spain
Hasso M et al. (2022) (Hasso et al., 2022)	R	78	43.60%	38^a^	All males	Ontario, Canada
Loconsole D et al. (2022) (Loconsole et al., 2022)	PO	10	100%	36.7	8 males (6 MSM) and 2 females	Southern Italy
Mailhe et al. (2022) (Mailhe et al., 2022)	PO	258	98%	35	Majority of males including MSM except 1 female and 1 transgender female	France
Nörz D et al. (2022) (Nörz et al., 2022)	PO	16	100%	37	All males (MSM)	Germany
Ouafi M et al. (2022) (Ouafi et al., 2023)	C/Sl	116	100%	37	All males (including mostly MSM) except 1 female	Northern France
Palich R et al. (2023) (Palich et al., 2023)	C-S	50	88%	34	All males including 49 MSM and 1 MSW	Paris, France
Peiró-Mestres A et al. (2022) (Peiró-Mestres et al., 2022)	PO	12	100%	38.5	All males (MSM)	Barcelona, Spain
Silva MST et al. (2022) (Silva et al., 2023)	PO	188	96.30%	33	All cisgender males (majority MSM) except 8 cisgender females	Brazil
Tarín-Vicente EJ et al. (2022) (Tarín-Vicente et al., 2022)	PO	180	99%	37	Majority of males including gay, bisexual, MSM except for a few heterosexual males or females	Madrid and Barcelona, Spain
Thornhill JP et al. (2022) (Thornhill et al., 2022b)	C-S	123	100%	34	Majority of trans females and cis females and five non-binary individuals	15 countries in WHO regions of Europe, the Americas, and Africa
Thornhill JP et al. (2022) (Thornhill et al., 2022a)	C-S	528	97%	38	All males except one trans or non-binary (heterosexual, homosexual, or bisexual)	16 countries in Europe, the Americas, Africa, Asia, and Australia
Ubals M et al. (2022) (Ubals et al., 2022)	PO	49	100%	33.5	All males	Spain
Veintimilla C et al. (2022) (Veintimilla et al., 2022)	PO	37	97%	31	All males (MSM)	Madrid, Spain

The prevalence of skin mpox viral DNA is presented in percentage. Age (years) is presented in median.

YOP, year of publication; n, number; DNA, deoxyribonucleic acid; MSM, men who have sex with men; MSW, men who have sex with women; %, percentage; C-S, case series; C/Sl, cross-sectional; PO, prospective observational; R, retrospective.

a Represents mean value.

### 3.2 General study characteristics

The baseline characteristics of the 14 studies included in the systematic review and meta-analysis consisting of three case series (Thornhill et al., 2022a; Thornhill et al., 2022b; Palich et al., 2023), two cross-sectional studies (García-Piqueras et al., 2022; Ouafi et al., 2023), eight prospective observational studies (Loconsole et al., 2022; Mailhe et al., 2022; Nörz et al., 2022; Peiró-Mestres et al., 2022; Tarín-Vicente et al., 2022; Ubals et al., 2022; Veintimilla et al., 2022; Silva et al., 2023), and one retrospective study (Hasso et al., 2022) are explained in **Table 1**. Most of the studies were conducted in mpox non-endemic countries such as Spain (5/14, 35.71%), France (3/14, 21.42%), Italy (1/14), Germany (1/14), Canada (1/14), and Brazil (1/14). On the other hand, 2 studies out of 14 were carried out in both mpox non-endemic and endemic countries at the same time, for example, approximately 15 countries in Europe, America, and Africa as well as 16 countries in Europe, Americas, Africa, Asia, and Australia, respectively (Thornhill et al., 2022a; Thornhill et al., 2022b). Most of the cases were adults above 18 years. The sample size of these included studies ranged from 10 (Loconsole et al., 2022) to as high as 528 (Thornhill et al., 2022a). Furthermore, 5 out of 14 studies reported travel history in mpox participants (García-Piqueras et al., 2022; Loconsole et al., 2022; Peiró-Mestres et al., 2022; Thornhill et al., 2022a; Silva et al., 2023). Though we searched for studies from any time, we only found reports from the current epidemic. In all the included studies, mpox was confirmed by diagnostic testing such as real-time PCR for mpox DNA. Most of the cases were men consisting mostly of MSM (men who have sex with men) in all the included studies except one case series study (Thornhill et al., 2022b) where the majority of the participants were women (cis females, trans females, and non-binary individuals). In the case series with the largest sample size by Thornhill et al., 96.4% (509 out of 528) of mpox-confirmed cases were MSM (Thornhill et al., 2022a). The common systemic symptoms or manifestations presented by most of the mpox patients comprised rash, fatigue, headaches, myalgia, fever, and lymphadenopathy, while others were tonsillitis, proctitis, pharyngitis, odynophagia, epiglottitis, and asthenia.

### 3.3 Summary measure and heterogeneity

**Figure 2:**
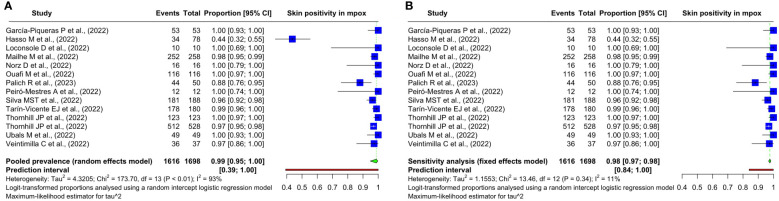
Forest plots showing the **(A)** overall pooled prevalence and its 95% confidence interval and heterogeneity statistics of the prevalence of mpox DNA in the skin samples of patients with mpox based on positivity rate (%) and **(B)** sensitivity analysis on the included studies.

A systematic review and meta-analysis of all 14 studies was carried out to assess the pooled prevalence of mpox viral DNA. Among the 1,754 confirmed mpox patients in the studies, skin samples were taken from 1,698, out of which 1,616 had mpox viral positivity in the skin samples. The pooled prevalence was 98.77% (95% CI: 94.74%–99.72%) using a random-effects model. There was significant heterogeneity in the results with an *I* ^2^ value of 92.5% (95% CI: 89.1%–94.8%). The studies show a relatively wide prediction interval of 39.22% to 99.99%. The individual study results, the methods used for the meta-analysis, and other results are summarized in **Figure 2A**.

### 3.4 Meta-regression

**Figure 3:**
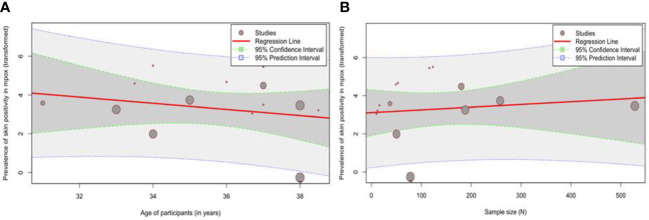
Bubble plots showing the meta-regression analysis of **(A)** age (years) and **(B)** sample size (*N*).

The bubble plots are produced after a simple meta-regression with continuous moderators and are shown in **Figure 3**. The effect size does not show a significant dependence on the moderator variables. The omnibus test of moderators yields *Q*_M_ values of 0.24 (*p* = 0.63) and 0.64 (*p* = 0.42) for meta-regression based on sample size and age, respectively.

### 3.5 Influence analysis

We detect outliers by observing the confidence interval (CI) of the individual study estimates. If the CI of a study does not overlap at all with the CI of the pooled effect, we declare the said study an outlier. In this case, one study (Hasso et al., 2022) fulfills this condition as can be observed in **Figure 2B**.

The Baujat plot shows that this study (Hasso et al., 2022) overly contributes to both the overall heterogeneity and the effect size of the summary estimate (**Supplementary Annex 4A**). In influence diagnostics (**Supplementary Annex 4B**), we see that the externally standardized residual of this study is more than four units away. The difference in fits suggests a considerable influence. Cook’s distance that depends both on residual and leverage also shows a high influence. A covariance ratio of even less than 1 indicates the need for the removal of the study for a sensitivity analysis. Here, the covariance ratio is less than 0.5 for this study. The leave-one-out *τ* ^2^ and Cochran’s *Q* plots show a massive dip in heterogeneity after excluding this study. The hat values and the study weight plots also indicate a considerable influence of this study. The leave-one-out meta-analysis sorted by *I* ^2^ shows a clear trend wherein *I* ^2^ varies between 91% and 93% for all the studies but is 10.8% for this case (**Supplementary Annex 4C**).

For the GOSH plots, instead of omitting one study at a time (as in the leave-one-out meta-analysis), we build meta-analytic models of all possible subsets of the included studies. Therefore, we have fit 8,192 meta-analytical models with the given studies and plotted the results (**Supplementary Annex 4D**). This helps identify clusters and highly influential studies. **Supplementary Annex 4E** shows the influence of this study (Hasso et al., 2022). All the fitted subsets are demonstrated, and the colored points correspond to only those models where this study was included. We can see that the presence of this study leads to a different cluster altogether. This cluster has much higher heterogeneity as indicated by the *I* ^2^ estimate in the *Y*-axis. Overall, we can easily conclude that this study is overly influential.

### 3.6 Sensitivity analysis

We found this study (Hasso et al., 2022) to be an overly influential outlier in our preceding analysis. So, we conducted a sensitivity analysis after excluding this study. The heterogeneity has dropped significantly. The previous *I* ^2^ of 92.5% (95% CI: 89.1%–94.8%) dropped to 10.8% (95% CI: 0.0%–49.7%). The prediction interval considerably narrowed from 39.22%–99.99% to 84.05%–99.90%. Given the homogeneity among these studies and a substantially decreased heterogeneity, we employed a common-effects model to meta-analyze the pooled prevalence in the sensitivity analysis. It has changed from 98.77% (95% CI: 94.74%–99.72%) to 97.65% (95% CI: 96.79%–98.29%). The findings are summarized in **Figure 2B**.

### 3.7 Publication bias

To assess small-study effects and publication bias, we calculated the LFK index performed in addition to the visual inspection of a Doi plot (**Supplementary Annex 5**). Most of the studies fall within the right limb. Moreover, the LFK index is 2.7 falling outside the limits of −1 to +1. This suggests asymmetry of the study findings.

### 3.8 Distribution of true effect size and prediction interval

**Figure 4:**
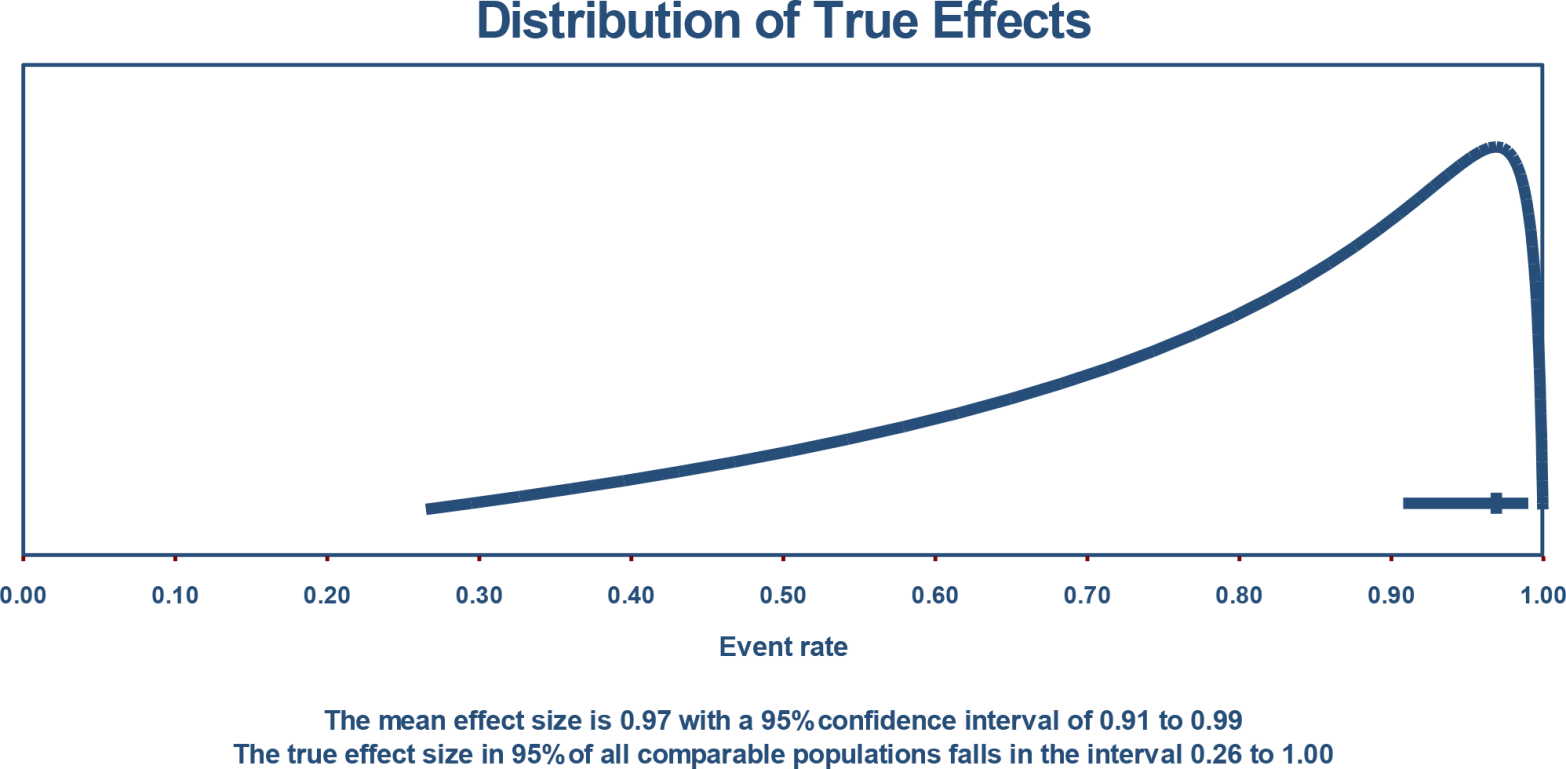
Distribution of true effect size computations.

If we assume that the true effects are normally distributed (in logit units), we can estimate that the prediction interval is 0.265 to 1.000. The true effect size in 95% of all comparable populations falls in this interval (**Figure 4**).

## 4 Discussion

The main result of this study is that the pooled proportion (%) of skin samples with mpox viral DNA was 98.77% (95% CI: 94.74%–99.72%) yielded from a total pool of 1,616 patients. To the best of our knowledge, the present systematic review and meta-analysis is the first study to evaluate the overall viral positivity rate in the cutaneous samples of mpox-infected cases.

Importantly, a few articles included in the meta-analysis have also mentioned a high viral load [lower cycle threshold (Ct) value] in the skin specimens of mpox-infected patients. The authors confirmed that a lower Ct value is predictive of a higher probability of skin samples being positive for mpox DNA. The current outcomes are also in concordance with a larger prospective study depicting a high viral burden in skin samples responsible for transmission which most likely occurs through direct body contact rather than through the respiratory route or contact with body fluids (Palich et al., 2023). Similarly, another *in vivo* study observed a remarkable correlation between viral DNA load and infectivity in the BSC-1 cell line with epithelial morphology. Moreover, an mpox-infected patient with lesions is considered infectious till the crust from the crusty lesions falls off (Ouafi et al., 2023). Notably, this extremely envisages a higher risk of transmission of infection from dermal lesions.

The results of this study also confirm earlier findings from a meta-analysis displaying high mpox viral load in skin samples than in other biological samples (Martins-Filho et al., 2022). The current data extends the previous results yielded from studies with a small sample size published through August 2022. We performed the meta-analysis on a large number of studies available worldwide till 17 January 2023 in order to assess the pooled proportion of mpox patients’ cutaneous specimens being positive with mpox viral DNA. Likewise, the current quantitative results considerably validated the high viral positivity rate in dermal specimens according to the latest several studies published worldwide. Especially, Noe et al. observed the highest viral concentrations (copy number/ml) in skin swabs of the first two mpox patients in Germany (Noe et al., 2023). These researchers were able to isolate mpox only from the skin pustules and proposed that skin (close) contact is the main route of transmission.

Furthermore, Suner and colleagues (Suñer et al., 2022) have revealed that skin lesions had a high median of viral DNA content of at least 2 orders of magnitude [7.3 log_10_ copies/ml (IQR 6.5–8.2)] compared with all the other clinical samples during the course of the disease. Moreover, the replication-competent viruses with high DNA levels (>6.5 log_10_ copies/ml) were isolated from dermal specimens. Moreover, these lesions had the longest median time [25 days (95% CI: 23–28)] of viral clearance from symptom onset than other clinical samples (Suñer et al., 2022).

Another longitudinal study on viral DNA load kinetics revealed that higher mpox viral load in skin lesion swabs was observed at the late stages of the disease. Despite this, all skin lesion samples were positive for mpox viral DNA during the entire time course as compared with the oropharyngeal samples (Nörz et al., 2022). Thus, contact transmission via mpox skin lesions may be a dominant route of mpox infection.

The current approach also highlights that skin lesion swabs are a suitable and reliable source of specimens for diagnostic purposes: they can be easily assessed using the real-time PCR technique. In line with these results, the WHO guidelines have recommended skin lesions as suitable diagnostic specimens for laboratory mpox confirmation (World Health Organization (WHO), 2022b; Jiang et al., 2022). Most of all, these samples are easy to collect from the roof or fluid from vesicles, pustules, and dry crusts of the skin lesions. A recent study has observed no statistically significant difference in the viral positivity rate of skin swabs among the self- or physician-collected samples (Ubals et al., 2022). This is suggestive of adopting self-sampling policies which will definitely benefit patients as well as disease control.

In the present study, Hasso and colleagues (Hasso et al., 2022) have reported that 43.6% of skin samples of mpox patients had mpox viral DNA. This study is observed to be an overly influential outlier during the preceding analysis, but this outlier only changes the pooled proportions from 98.77% (95% CI: 99.74%–99.72%) to 97.65% (95% CI: 96.79%–98.29%). However, Hasso et al. (2022) observed that skin lesions were most frequently positive (92.3%) in mpox patients who were analyzed for >1 skin specimen than other samples, indicating that testing multiple skin samples may increase the sensitivity of this test.

Taken alongside the data from earlier studies, our study suggests that skin lesions can play a main role in the transmission of mpox, either directly through cutaneous contact or indirectly through contaminated materials. Our data might be translated into informed decision-making regarding guidelines for mpox patients and for preventive as well as containment measures and can be implemented to prevent the spread of the infection in multinational outbreaks. Notably, this study has some shortcomings such as the restricted sample size or the limited number of selected studies. Preferably, a bigger sample size would assure good accuracy in records assessment. However, the current outcomes are based on consistent literature; thus, they appear reliable. Furthermore, the correlation of skin viral positivity rate with a high risk of infectivity potential is established upon the particular features of mpox infection. For example, most of the included studies had male mpox cases of all ages mainly from non-endemic regions. It would be a better method to emphasize the role of cutaneous viral burden in the severity and infectivity of illness according to age, gender, and endemic and non-endemic regions. Another limitation involves the lack of data about testing samples from multiple sites such as mucocutaneous or body fluids, and facts from these clinical specimens may improve diagnostic sensitivity and reduce false-negative test results. Indeed, extensive studies are required to attain a logical understanding of transmission such as factors that have allowed the surprising penetration of active mpox infection into human communities globally.

## 5 Conclusion

The present study provides an estimate of the pooled positivity rate of skin samples from mpox patients. It provides novel and reliable evidence regarding the potential role of direct skin-to-skin contact in mpox transmission, relating to a high risk of transmission of infection from dermal lesions. This new knowledge can allow focusing on mitigation and containment measures to flatten the peak of mpox infection during future spreads.

## Author contributions

Substantial contributions to the conception or design of the work: BKP, IR, AG, MAS, PS, and JJB. Acquisition, analysis, or interpretation of data for the work: BKP, MAS, IR, and AG. Drafting the work: IR, AG, PS, MAS, and BKP. Revising the manuscript critically for important intellectual content: RS, BKP, AP, KG, RoS, IR, AG, and JJB. Final approval of the version to be published: all authors (IR, PS, AG, MAS, AP, RoS, KG, RS, JJB, and BKP). Agreement to be accountable for all aspects of the work in ensuring that questions related to the accuracy or integrity of any part of the work are appropriately investigated and resolved: all authors (IR, PS, AG, MAS, AP, RoS, KG, RS, JJB, and BKP).

## Supplementary material

Supplementary Annex 1 Inclusion and Exclusion Criteria according to PECOS.

Supplementary Annex 2 The adjusted search terms as per the PECOS framework (prevalence of mpox DNA in skin samples of patients with monkeypox virus infection): searched electronic databases [as of 17.01.2023].

Supplementary Annex 3 Risk of Bias assessment of included studies using NIH tools **(A)** for Case series; **(B)** Cross-sectional studies.

Supplementary Annex 4 Influence analysis for confirming the meta-analysis conducted for the prevalence of mpox viral DNA in skin samples: **(A)** Baujat plot showing the studies which overly contribute to the overall heterogeneity, **(B)** Influence diagnostics, **(C)** Leave-one-out study method sorted by I^2^, **(D)** GOSH plot showing the estimate and heterogeneity of meta-analysis of all possible subsets of included studies, **(E)** GOSH plot showing the contributions of the influential outlying study.

Supplementary Annex 5 Doi plot and LFK index to show asymmetry of study findings.
