# Supplementary material for: Prevalence of mpox viral DNA in cutaneous specimens of monkeypox-infected patients: a systematic review and meta-analysis
Source: Front Cell Infect Microbiol. 2023 Jun 29;13:1179885. doi: 10.3389/fcimb.2023.1179885 (PMC10349178; doi:10.3389/fcimb.2023.1179885)
Supplement: Supplementary Annex 1 — Inclusion and Exclusion Criteria according to PECOS. [file DataSheet_1.docx]

**Annex 1: Inclusion and exclusion criteria according to PECOS**

| **Participants** | **Exposure** | **Comparator** | **Outcome** | **Study design** |
| --- | --- | --- | --- | --- |
| **Inclusion criteria:**  All confirmed mpox patients by real time PCR   - All ages - All gender   **Exclusion criteria:**  Suspected or probable mpox patients | Not applicable | Not applicable | **Inclusion criteria:**  Frequency of skin samples positive with mpox viral DNA | **Inclusion criteria:**  Observational studies, Prevalence studies, case series, cross-sectional studies, cohort studies, case control studies, surveys, preprints, Editorial with case series data, Rapid or Short communication with case series data, brief reports with case series data.  Geography-Global level.  Date of Search- Published till 17^th^ January 2022. English Language. Human studies.  **Exclusion criteria:**  Qualitative, Policy, case reports, Opinion reports, abstracts |

**Annex 2: The adjusted search terms as per the PECOS framework (prevalence of mpox DNA in skin samples of patients with monkeypox virus infection): searched electronic databases [as of 17.01.2023]**

| **Database** | **No** | **Search Query** | **Results** |
| --- | --- | --- | --- |
| **Cochrane** | | | |
|  | #1 | (mpox:ti,ab) OR (monkeypox:ti,ab) OR (mpxv:ti,ab) | 14 |
|  | #2 | (skin:ti,ab) OR (cutaneous*:ti,ab) | 61,030 |
|  | #3 | (lesion*:ti,ab) OR (swab:ti,ab) OR (sample:ti,ab) | 145,138 |
|  | #4 | #1 AND #2 AND #3 | 3 |
| **EBSCOHost-Academic Search Complete** | | | |
|  | #1 | ((TI mpox OR AB mpox)) OR ((TI monkeypox OR AB monkeypox)) OR ((TI mpxv OR AB mpxv)) | 104 |
|  | #2 | ((TI skin OR AB skin)) OR ((TI cutaneous* OR AB cutaneous*)) | 19,016 |
|  | #3 | ((TI lesion* OR AB lesion*)) OR ((TI swab OR AB swab)) OR ((TI sample OR AB sample)) | 134,813 |
|  | #4 | #1 AND #2 AND #3 | 4 |
| **EMBASE** | | | |
|  | #1 | (mpox:ti,ab) OR (monkeypox:ti,ab) OR (mpxv:ti,ab) | 2,577 |
|  | #2 | (skin:ti,ab) OR (cutaneous*:ti,ab) | 985,816 |
|  | #3 | (lesion*:ti,ab) OR (swab:ti,ab) OR (sample:ti,ab) | 2,660,413 |
|  | #4 | #1 AND #2 AND #3 | 162 |
| **ProQuest** | | | |
|  | #1 | (TI,AB(mpox)) OR (TI,AB(monkeypox)) OR (TI,AB(mpxv)) | 564 |
|  | #2 | (TI,AB(skin)) OR (TI,AB(cutaneous*)) | 109,047 |
|  | #3 | (TI,AB(lesion*)) OR (TI,AB(swab)) OR (TI,AB(sample)) | 1,135,291 |
|  | #4 | #1 AND #2 AND #3 | 42 |
| **PubMed** | | | |
|  | #1 | (mpox[Title/Abstract]) OR (monkeypox[Title/Abstract]) OR (mpxv[Title/Abstract]) OR ("monkeypox"[MeSH]) | 2,457 |
|  | #2 | ("skin"[MeSH Terms]) OR (skin[Title/Abstract]) OR (cutaneous*[Title/Abstract]) | 812,419 |
|  | #3 | (lesion*[Title/Abstract]) OR (swab[Title/Abstract]) OR (sample[Title/Abstract]) | 1,949,255 |
|  | #4 | #1 AND #2 AND #3 | 173 |
| **Scopus** | | | |
|  | #1 | (TITLE-ABS(mpox)) OR (TITLE-ABS(monkeypox)) OR (TITLE-ABS(mpxv)) | 2,400 |
|  | #2 | (TITLE-ABS(skin)) OR (TITLE-ABS(cutaneous*)) | 1,006,876 |
|  | #3 | (TITLE-ABS(lesion*)) OR (TITLE-ABS(swab)) OR (TITLE-ABS(sample)) | 6,140,713 |
|  | #4 | #1 AND #2 AND #3 | 145 |
| **Web of Science** | | | |
|  | #1 | ((TI=mpox OR AB=mpox)) OR ((TI=monkeypox OR AB=monkeypox)) OR ((TI=mpxv OR AB=mpxv)) | 1,729 |
|  | #2 | ((TI=skin OR AB=skin)) OR ((TI=cutaneous* OR AB=cutaneous*)) | 664,566 |
|  | #3 | ((TI=lesion* OR AB=lesion*)) OR ((TI=swab OR AB=swab)) OR ((TI=sample OR AB=sample)) | 4,262,363 |
|  | #4 | #1 AND #2 AND #3 | 100 |

**Annexure 3: Risk of Bias assessment of included studies using NIH tools (A) for Case series; (B) Cross-sectional studies**

**(A)**

| **Author (YOP)** | **Q1** | **Q2** | **Q3** | **Q4** | **Q5** | **Q6** | **Q7** | **Q8** | **Q9** | **Overall Quality** |
| --- | --- | --- | --- | --- | --- | --- | --- | --- | --- | --- |
| **Loconsole D et al., (2022)** (37) | Y | Y | Y | NA | NA | Y | NA | Y | Y | Good |
| **Palich R et al., (2023)** (15) | Y | Y | Y | NA | NA | Y | NA | Y | Y | Good |
| **Peiró-Mestres A et al., (2022)** (16) | Y | Y | Y | NA | NA | Y | NA | Y | Y | Good |
| **Thornhill JP et al., (2022)** (31) | Y | Y | Y | NA | NA | Y | NA | Y | Y | Good |
| **Thornhill JP et al., (2022)** (9) | Y | Y | Y | NA | NA | Y | NA | Y | Y | Good |

YOP: Year of Publication; Y: Yes; N: No; NA: Not Applicable; CD: Cannot Determine; NI: No Information; NIH: National Institute of Health

Q1: Was the study question or objective clearly stated?
Q2: Was the study population clearly and fully described, including a case definition?
Q3: Were the cases consecutive?
Q4: Were the subjects comparable?
Q5: Was the intervention clearly described?
Q6: Were the outcome measures clearly defined, valid, reliable, and implemented consistently across all study participants?
Q7: Was the length of follow-up adequate?
Q8: Were the statistical methods well-described?
Q9: Were the results well-described?

**(B)**

| **Author (YOP)** | **Q1** | **Q2** | **Q3** | **Q4** | **Q5** | **Q6** | **Q7** | **Q8** | **Q9** | **Q10** | **Q11** | **Q12** | **Q13** | **Q14** | **Quality rating** |
| --- | --- | --- | --- | --- | --- | --- | --- | --- | --- | --- | --- | --- | --- | --- | --- |
| **García-Piqueras P et al. (2022) (32)** | Y | Y | Y | Y | N | NA | NA | NA | NA | NA | Y | NA | NA | NA | Good |
| **Hasso M et al. (2022) (41)** | Y | Y | Y | Y | N | NA | NA | NA | NA | NA | Y | NA | NA | NA | Good |
| **Mailhe et al. (2022) (35)** | Y | Y | Y | Y | N | NA | NA | NA | NA | NA | Y | NA | NA | NA | Good |
| **Nörz D et al. (2022) (39)** | Y | Y | Y | Y | N | NA | NA | NA | NA | NA | Y | NA | NA | NA | Good |
| **Ouafi M et al. (2022) (33)** | Y | Y | Y | Y | N | NA | NA | NA | NA | NA | Y | NA | NA | NA | Good |
| **Silva MST et al. (2022) (40)** | Y | Y | Y | Y | N | NA | NA | NA | NA | NA | Y | NA | NA | NA | Good |
| **Tarín-Vicente EJ et al. (2022) (36)** | Y | Y | Y | Y | N | NA | NA | NA | NA | NA | Y | NA | NA | NA | Good |
| **Ubals M et al. (2022) (34)** | Y | Y | Y | Y | N | NA | NA | NA | NA | NA | Y | NA | NA | NA | Good |
| **Veintimilla C et al. (2022) (38)** | Y | Y | Y | Y | N | NA | NA | NA | NA | NA | Y | NA | NA | NA | Good |

YOP: Year of Publication; Y: Yes; N: NO; NA: Not Applicable; CD: Cannot Determine; NI: No Information; NIH: National Institute of Health

Q1: Was the research question or objective in this paper clearly stated?
Q2: Was the study population clearly specified and defined?
Q3: Was the participation rate of eligible persons at least 50%?
Q4: Were all the subjects selected or recruited from the same or similar populations (including the same time period)? Were inclusion and exclusion criteria for being in the study prespecified and applied uniformly to all participants?
Q5: Was a sample size justification, power description, or variance and effect estimates provided?
Q6: For the analyses in this paper, were the exposure(s) of interest measured prior to the outcome(s) being measured?
Q7: Was the timeframe sufficient so that one could reasonably expect to see an association between exposure and outcome if it existed?
Q8: For exposures that can vary in amount or level, did the study examine different levels of the exposure as related to the outcome (e.g., categories of exposure, or exposure measured as continuous variable)?
Q9: Were the exposure measures (independent variables) clearly defined, valid, reliable, and implemented consistently across all study participants?
Q10: Was the exposure(s) assessed more than once over time?
Q11: Were the outcome measures (dependent variables) clearly defined, valid, reliable, and implemented consistently across all study participants?
Q12: Were the outcome assessors blinded to the exposure status of participants?
Q13: Was loss to follow-up after baseline 20% or less?
Q14: Were key potential confounding variables measured and adjusted statistically for their impact on the relationship between exposure(s) and outcome(s)?

**Annexure 4:** Influence analysis for confirming the meta-analysis conducted for the prevalence of mpox viral DNA in skin samples: **(A)** Baujat plot showing the studies which overly contribute to the overall heterogeneity, **(B)** Influence diagnostics**, (C)** Leave-one-out study method sorted by I^2^, **(D)** GOSH plot showing the estimate and heterogeneity of meta-analysis of all possible subsets of included studies, **(E)** GOSH plot showing the contributions of the influential outlying study

**(A)**

**
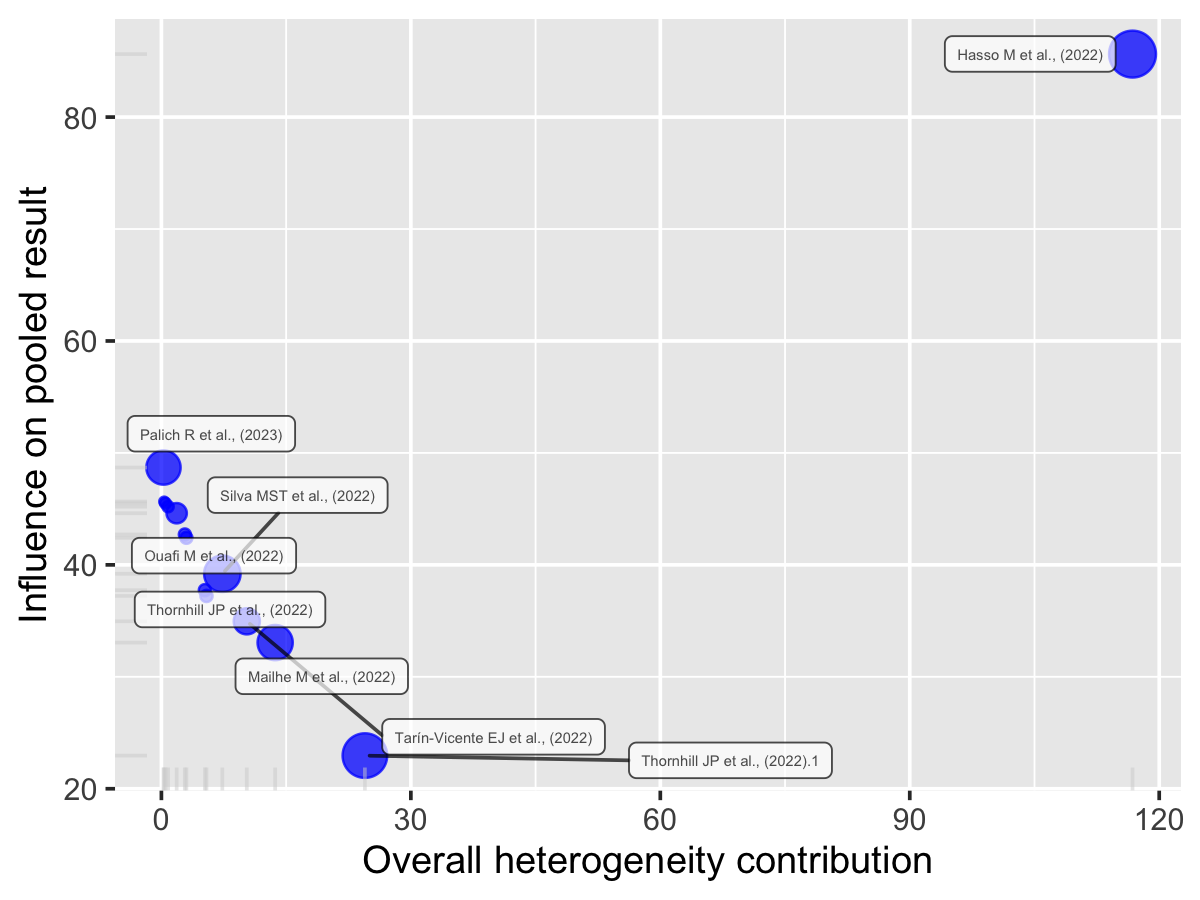
**

**(B)**

**
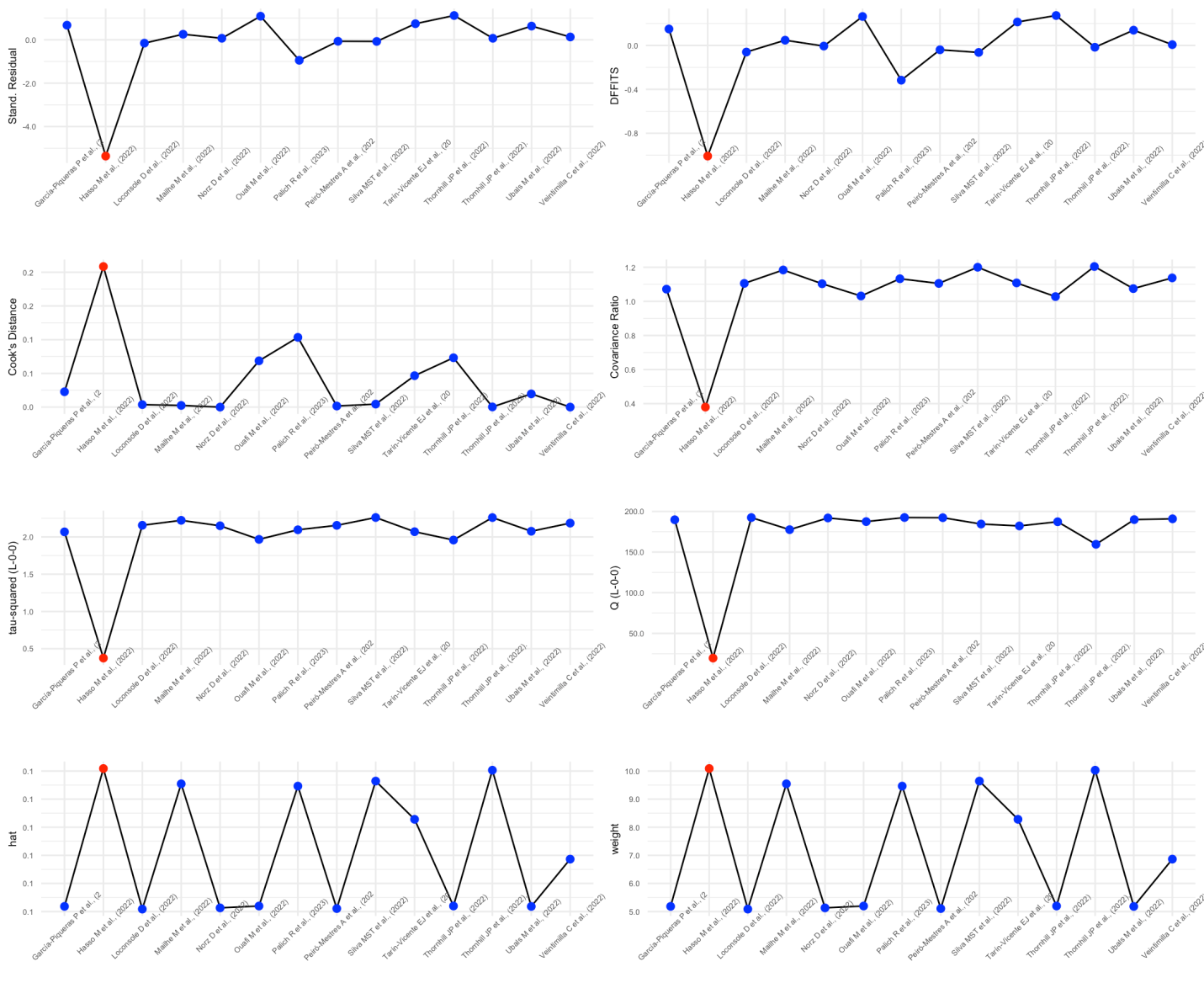
**

**(C)**

**
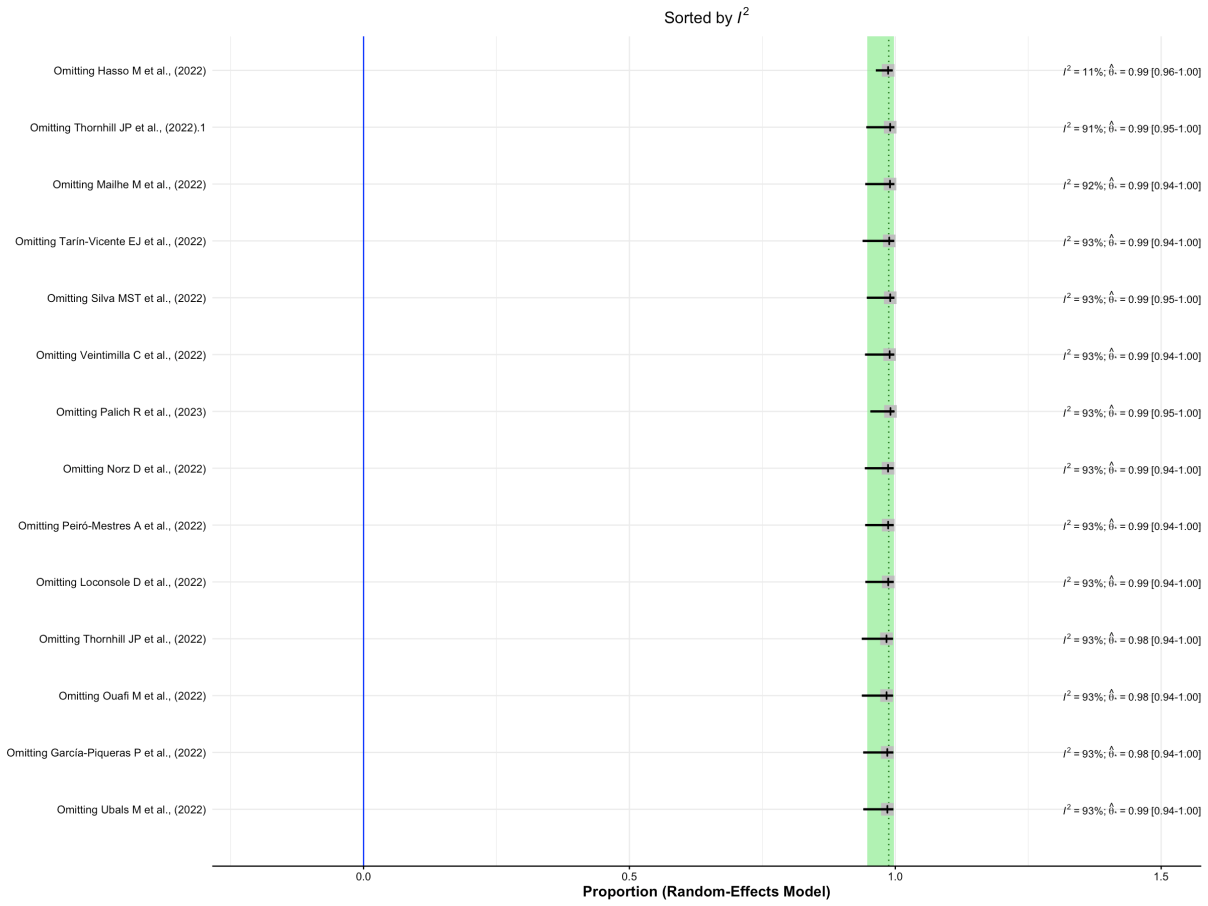
**

**(D)**

**
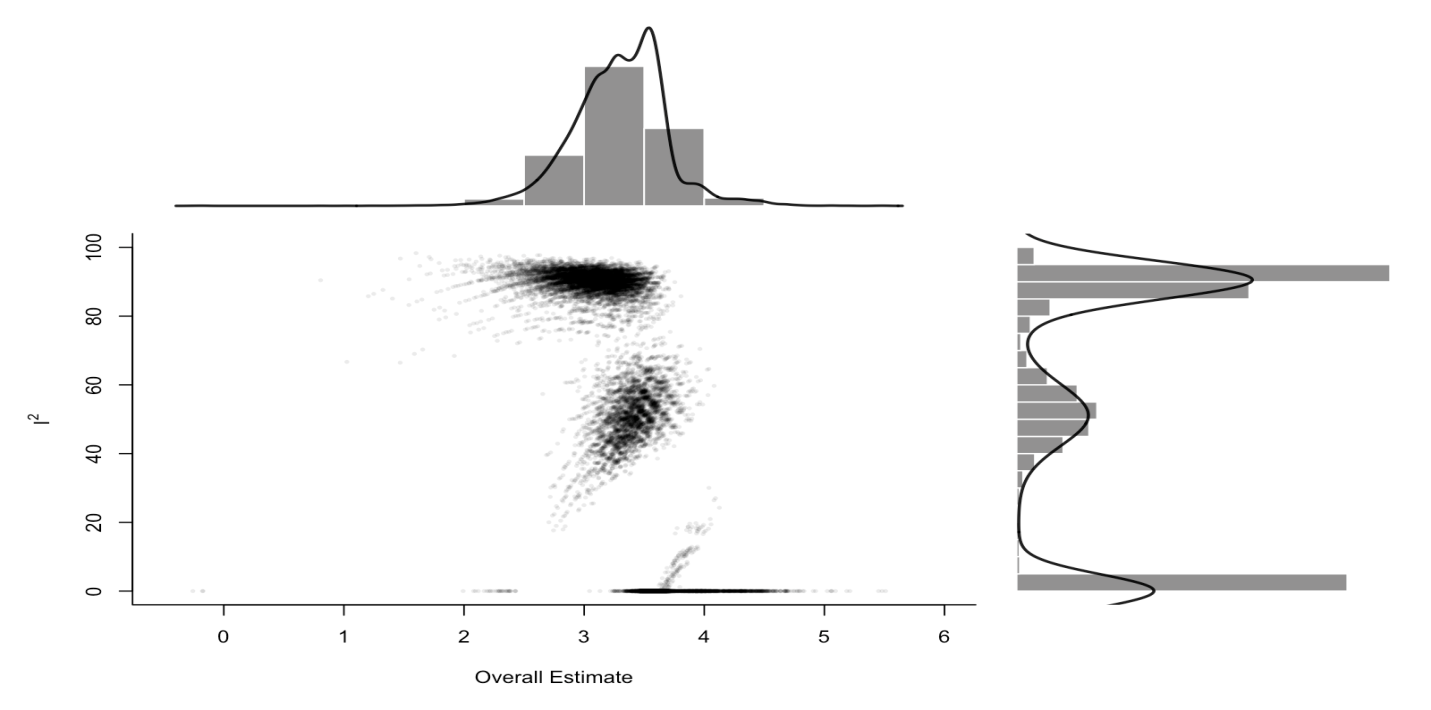
**

**(E)**

**
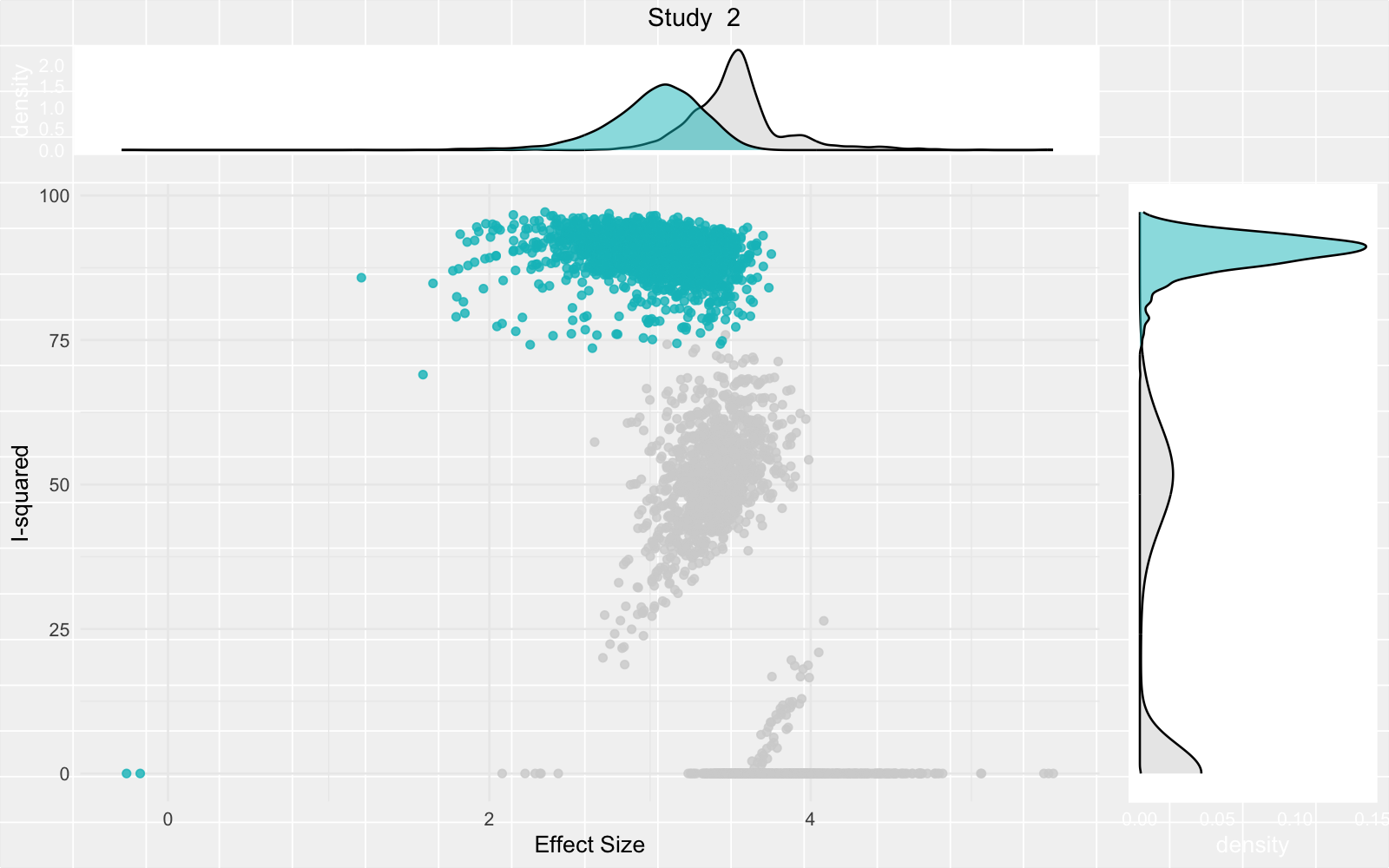
**

**Annexure 5:** Doi plot and LFK index to show asymmetry of study findings

**
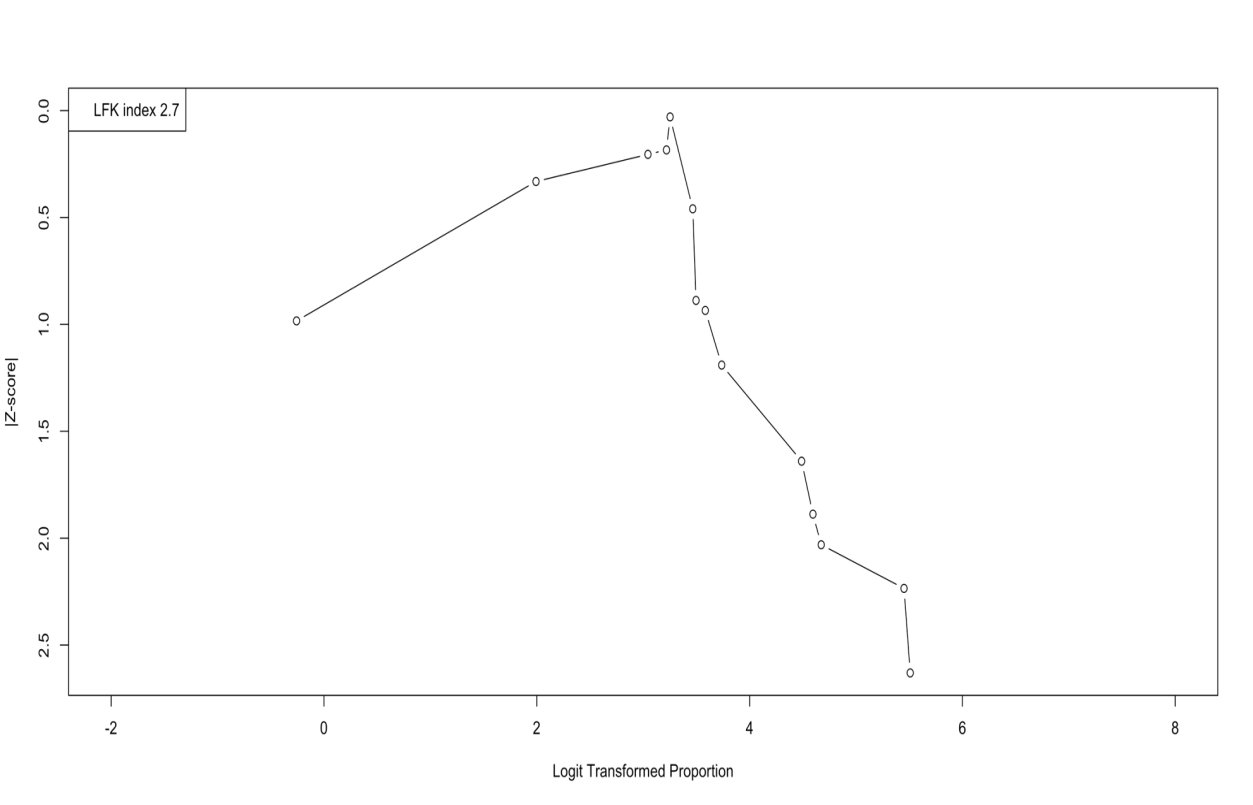
**

**
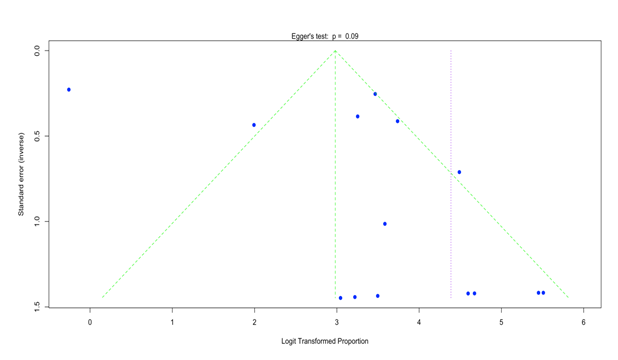
**
